# Chitosan attenuates titanium dioxide nanoparticles induced hepatic and renal toxicities

**DOI:** 10.1038/s41598-025-01736-2

**Published:** 2025-05-30

**Authors:** Amal Abdelmonem Halawa, Gehad Elshopakey, Mohamed El-Adl, Samah Lashen, Nancy Shalaby, Shaymaa Rezk, Omar Elmetwally, Ehab Eldomany, Ahmed Farghali, Mohamed Elmetwally

**Affiliations:** 1 Department of Forensic Medicine and Toxicology, Faculty of Veterinary Medicine, Mansoura University, Mansoura, 35516 Egypt; 2 Department of Clinical Pathology, Faculty of Veterinary Medicine, Mansoura University, Mansoura, 35516 Egypt; 3 Department of Biochemistry and Molecular Biology, Faculty of Veterinary Medicine, Mansoura University, Mansoura, 35516 Egypt; 4 Department of Cytology and Histology, Faculty of Veterinary Medicine, Mansoura University, Mansoura, 35516 Egypt; 5 Department of Forensic Medicine and Clinical Toxicology, Faculty of Medicine, Damietta University, Damietta, 34517 Egypt; 6 Department of Internal Medicine, Hepatology and Gastroenterology Unit, Faculty of Medicine, Mansoura University, Mansoura, 35516 Egypt; 7 Department of Biotechnology and life sciences, Faculty of Postgraduate Studies for Advanced Sciences (PSAS), Beni-Suef University, Beni-Suef, 62511 Egypt; 8 Department of Material Science and Nanotechnology, Faculty of Postgraduate Studies for Advanced Sciences (PSAS), Beni-Suef University, Beni-Suef, 62511 Egypt; 9 Department of Theriogenology, Faculty of Veterinary Medicine, Mansoura University, Mansoura, 35516 Egypt

**Keywords:** Nanotoxicology, Titanium dioxide nanoparticles, Chitosan, Biomarkers, Health care

## Abstract

Titanium dioxide nanoparticles (TiO_2_ NPs) are extensively incorporated in numerous industrial products. Adult male Albino rats received oral TiO_2_ NPs at a dose of 150 mg/kg body weight for 14 days exhibited both hepatic and renal toxicities manifested by disruption in serum hepatic and renal biomarkers, imbalance in oxidative-antioxidant system, up-regulation of mRNA expression of genes encode inflammation (IL-1β, TNF-α) and apoptosis (Caspase-3, BAX) with down-regulation of PCNA immune-staining density and histological modifications in hepatic and renal architecture. Carboxymethyl chitosan (5 mg/kg BW) significantly improved the harmful effects of nano-titanium particles highlighting its relevance in reducing TiO_2_ NPs – induced hepatic and renal dysfunction.

## Introduction

They resemble a scientific revolution that changes the properties of various compounds creating a new element with new characteristics and effects. Nanoparticles, their small size and large surface area afford them with an intrinsic toxicity or active group and their impact on humans, animals and environment is a critical issue. One of the most extensively distributed and highly utilized nanoparticles worldwide are titanium dioxide nanoparticles (TiO_2_ NPs) which are integrated in abundant industries such as food, medical and cosmetic products. It has been used for the manufacture of coated candy, carbonated drinks, chewing gum, preserved fruits, milk and dairy products, and other food categories. The concentration of nano-titanium in food ranges as high as 0.5–9 g/kg^1–3^. Following ingestion, the most common route of exposure, TiO_2_ NPs can be absorbed through gastrointestinal tract and deposited in different organs like liver, kidney, brain, lung, spleen, and heart^4^. Hepatic and renal toxicities of nano-titanium particles were previously studied via different exposure routes^4–6^. In liver and kidney toxicities, several stress factors prompt the production of reactive oxygen species, with subsequent elevation in the levels of malondialdehyde, total protein, numerous inflammatory markers and organ-specific biomarkers that may encourage mitochondrial dysfunction^7^. Intraperitoneal TiO_2_ NPs at doses of 150 and 200 mg/kg for 14 days elevated alkaline phosphatase, alanine aminotransferase and aspartate aminotransferase enzymes, albumin, total bilirubin, and total protein levels with declines in serum levels of uric acid and blood urea nitrogen signifying hepatic and renal dysfunctions^8,9^. Moreover, treatment of mice with 10 or 50 mg/kg TiO_2_ NPs for 60 days resulted in significant surges in the levels of hydrogen peroxide, nitric oxide and malondialdehyde, while hepatic mRNA levels of SOD, catalase, GST and GSHPx genes were decreased^9^. Rats received 300 mg/kg of TiO_2_ NPs for 14 days by gavage method exhibited hepatic oxidative stress through the increase in lipid peroxidation and diminution in GPx and SOD enzymes with an increase in apoptotic index^10^. Chitosan is a linear polysaccharide that is prepared by treating the chitin shells of shrimp as well as other crustaceans with an alkaline material. Owing to its distinctive physical and chemical properties, chitosan has a varied array of applications in the medical field. Chitosan has received tremendous attention as a biomedical material for its numerous biomedical activities, including anticancer, antioxidant, anticoagulant, immune-stimulating, and anti-inflammatory properties^11,12^.

The liver is the main site for drug and xenobiotic metabolism. While the renal system is imperative for elimination of toxic wastes from the body. Therefore, both organs are critical in protecting the body from the adverse effects of harmful chemicals. Inspecting the previous findings on the biological activities of chitosan, it is rational to speculate that chitosan may possess a significant anti-TiO_2_ NPs effect. Therefore, in the present work, we investigated hepatic and renal toxicity of oral TiO_2_ NPs as well as the possible improvement of such effects by oral administration of chitosan, with possible underlying mechanism of action.

## Results

### Characterization of TiO_2_ NPs

**Fig. 1:**
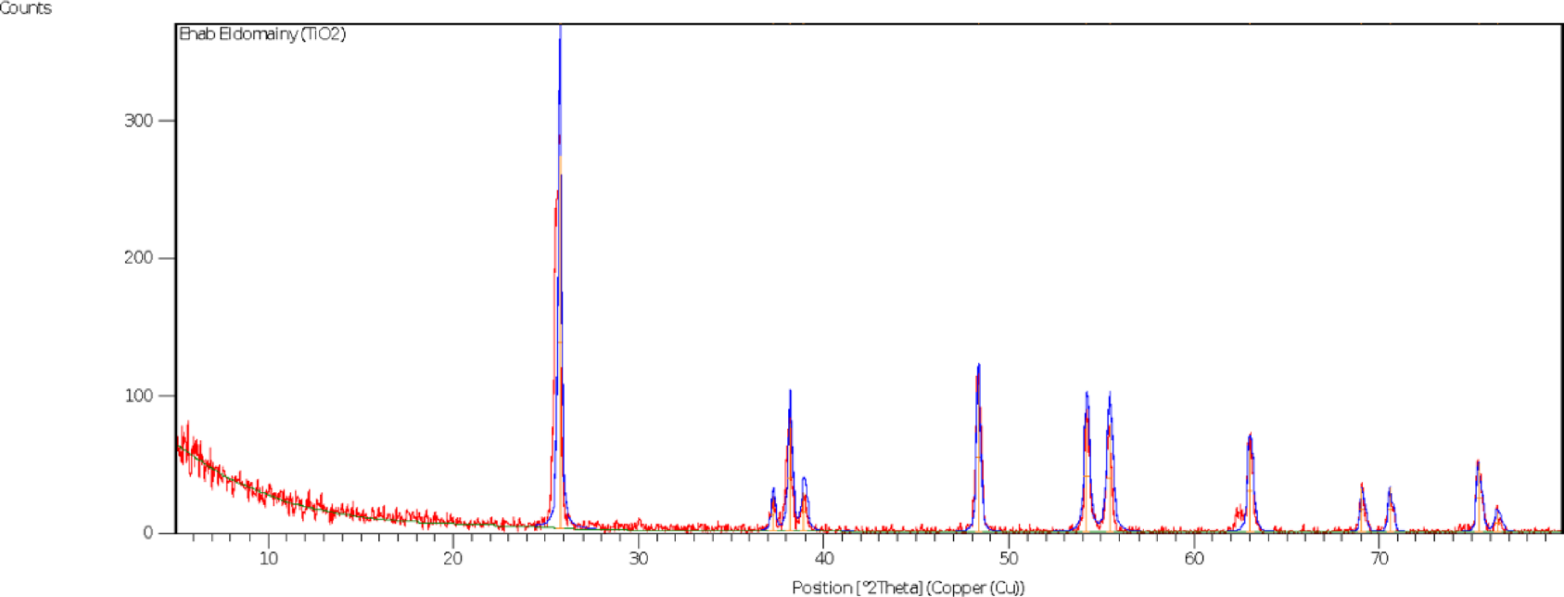
Characterization of TiO_2_ nanoparticles, X-ray diffraction (XRD) peak of crystalline powder of TiO_2_.

Nano titanium dioxide particles were prepared by ball milling, the crystalline powder of TiO_2_ was confirmed by X-Ray-Diffraction (XRD). The size of TiO_2_ NPs was average 50–55 nm (Fig. 1).

### Relative weights of liver and kidney

After 14 days, no apparent difference was observed in relative weights of either livers or kidneys among experimental groups (Table 1).

**Table 1:** Effects of TiO_2_ NPs and Chitosan on relative weights of liver and kidney (g).

	Experimental groups			
	Control	Ch	TiO_2_-NPs	TiO_2_-NPs + Ch
**Liver**	2.117 ± 0.759	3.273 ± 0.139	3.424 ± 0.130	2.834 ± 0.034
**Kidney**	0.248 ± 0.058	0.352 ± 0.016	0.357 ± 0.008	0.282 ± 0.080

### Serum hepatic and renal biomarkers

Activities of ALT, AST, and ALP were significantly elevated in TiO_2_ NPs group with increasing levels of total, direct and indirect bilirubin compared to the control group (*p* < 0.05). Moreover, the serum proteinogram assay showed a significant decrease of total protein, albumin, and globulin levels in TiO_2_ NPs group relative to the control one (*p* < 0.05). Likewise, renal toxicity was noticed following exposure to TiO_2_ NPs, indicated by significant upsurge in urea, creatinine, and uric acid levels (*p* < 0.05). Interestingly, the concomitant chitosan treatment significantly ameliorated nano-titanium induced hepatic and renal damage as demonstrated in TiO_2_ NPs + Ch group (*p* < 0.05) (Table 2).

**Table 2:** Serum levels of hepatic and renal biomarkers.

Parameters	Experimental groups			
	Control	Ch	TiO_2_-NPs	TiO_2_-NPs + Ch
*ALT (U/L)*	27.26 ± 1.52^c^	30.85 ± 1.55^c^	56.61 ± 2.78^a^	43.54 ± 1.72^b^
*AST (U/L)*	53.56 ± 2.86^c^	47.16 ± 2.23^c^	83.28 ± 2.49^a^	70.21 ± 1.85^b^
*ALP (U/L)*	260.99 ± 14.59^c^	258.04 ± 21.6^c^	788.54 ± 43.01^a^	591.51 ± 31.94^b^
*Total protein (g/dL)*	6.07 ± 0.22^a^	5.73 ± 0.18^a^	3.34 ± 0.20^c^	4.91 ± 0.13^b^
*Albumin (g/dL)*	3.14 ± 0.16^a^	2.89 ± 0.15^a^	2.03 ± 0.12^b^	2.41 ± 0.05^b^
*Globulin (g/dL)*	2.93 ± 0.21^a^	2.84 ± 0.16^a^	1.31 ± 0.11^b^	2.49 ± 0.10^a^
*Total bilirubin (mg/dL)*	0.38 ± 0.2^c^	0.34 ± 0.05^c^	0.67 ± 0.02^a^	0.47 ± 0.01^b^
*Direct bilirubin (mg/dL)*	0.22 ± 0.01^c^	0.19 ± 0.02^c^	0.38 ± 0.01^a^	0.29 ± 0.01^b^
*Indirect bilirubin (mg/dL)*	0.16 ± 0.01^b^	0.15 ± 0.03^b^	0.29 ± 0.02^a^	0.18 ± 0.02^b^
*Urea (mg/dL)*	57.95 ± 2.26^c^	63.52 ± 2.11^c^	90.61 ± 2.24^a^	80.64 ± 3.14^b^
*Creatinine (mg/dL)*	0.51 ± 0.03^c^	0.62 ± 0.02^c^	1.43 ± 0.13^a^	0.88 ± 0.03^b^
*Uric acid (mg/dL)*	3.07 ± 0.23^c^	2.72 ± 0.54^c^	7.28 ± 0.39^a^	5.40 ± 0.48^b^

Data are expressed as mean ± SEM (*n* = 5). Means in the same row with different superscripts are significantly different (*p* < 0.05). ALT: Alanine aminotransferase; AST: Aspartate aminotransferase; ALP: alkaline phosphatase.

### Oxidative/Antioxidant biomarkers

TiO_2_ NPs induced hepatic as well as renal oxidative stress through elevation of MDA content relative to the control group (*p* < 0.05). Moreover, TiO_2_ NPs impaired antioxidant complex activities via reducing hepatic as well as renal GSH, catalase, GPx, GST, and SOD levels compared to the control group (*p* < 0.05). Furthermore, chitosan treatment significantly amended the hepatic antioxidant complex and renal levels of MDA, GSH, catalase, and SOD toward the normal level when compared to both TiO_2_ NPs and control groups (*p* < 0.05). Considerable enhancement of the examined antioxidants proteins (hepatic and renal catalase, renal SOD, and hepatic GSH levels) was observed with depleting the pro-oxidants (hepatic MDA level) in chitosan administered group compared to control (*p* < 0.05) (Table 3).

**Table 3:** Hepatic and renal antioxidant/oxidative stress biomarkers.

Parameters	Experimental groups			
	Control	Chitosan	TiO_2_ NPs	TiO_2_NPs + Ch
*Liver*				
MDA(nmol/g tissue)	28.49 ± 2.07^c^	21.46 ± 2.13^d^	46.10 ± 2.42^a^	36.95 ± 1.39^b^
Catalase (U/g tissue)	5.10 ± 0.28^b^	7.24 ± 0.23^a^	2.84 ± 0.11^d^	3.64 ± 0.08^c^
GSH (mg/g tissue)	5.27 ± 0.33^b^	7.91 ± 0.29^a^	2.53 ± 0.24^d^	3.78 ± 0.22^c^
GPx (U/g tissue)	395.36 ± 9.92^ab^	422.58 ± 18.55^a^	324.84 ± 4.81^c^	370.48 ± 8.82^b^
GST (U/g tissue)	26.01 ± 0.99^a^	26.31 ± 1.52^a^	15.16 ± 1.49^c^	21.81 ± 0.92^b^
SOD (U/g tissue)	548.95 ± 17.05^a^	576.04 ± 28.03^a^	316.50 ± 24.24^c^	415.12 ± 21.65^d^
*Kidney*				
MDA(nmol/g tissue)	30.68 ± 1.24^c^	26.55 ± 2.03^c^	55.13 ± 2.50^a^	44.36 ± 1.12^b^
Catalase (U/g tissue)	2.52 ± 0.12^b^	3.92 ± 0.32^a^	1.09 ± 0.04^d^	1.71 ± 0.11^c^
GSH (mg/g tissue)	4.94 ± 0.19^a^	4.62 ± 0.14^a^	1.72 ± 0.37^c^	2.91 ± 0.28^b^
GPx (U/g tissue)	319.86 ± 3.93^a^	344.83 ± 10.72^a^	197.29 ± 10.85^b^	213.91 ± 15.97^b^
GST (U/g tissue)	16.51 ± 1.24^a^	17.42 ± 1.34^a^	9.56 ± 0.92^b^	12.85 ± 1.08^b^
SOD (U/g tissue)	358.87 ± 11.92^b^	390.31 ± 6.49^a^	218.162 ± 9.04^d^	277.74 ± 7.53^c^

Data were expressed as mean ± SEM (*n* = 5). Means in the same row with different superscripts are significantly different (*p* < 0.05). MDA, Malondialdehyde; GSH, Glutathione; GPx, Glutathione peroxidase; GST, Glutathione-S-transferase, SOD, Superoxide dismutase.

### Effects of TiO_2_ NPs and Chitosan on relative expression of hepatic genes encode apoptosis and inflammation (Fig. 2)

**Fig. 2:**
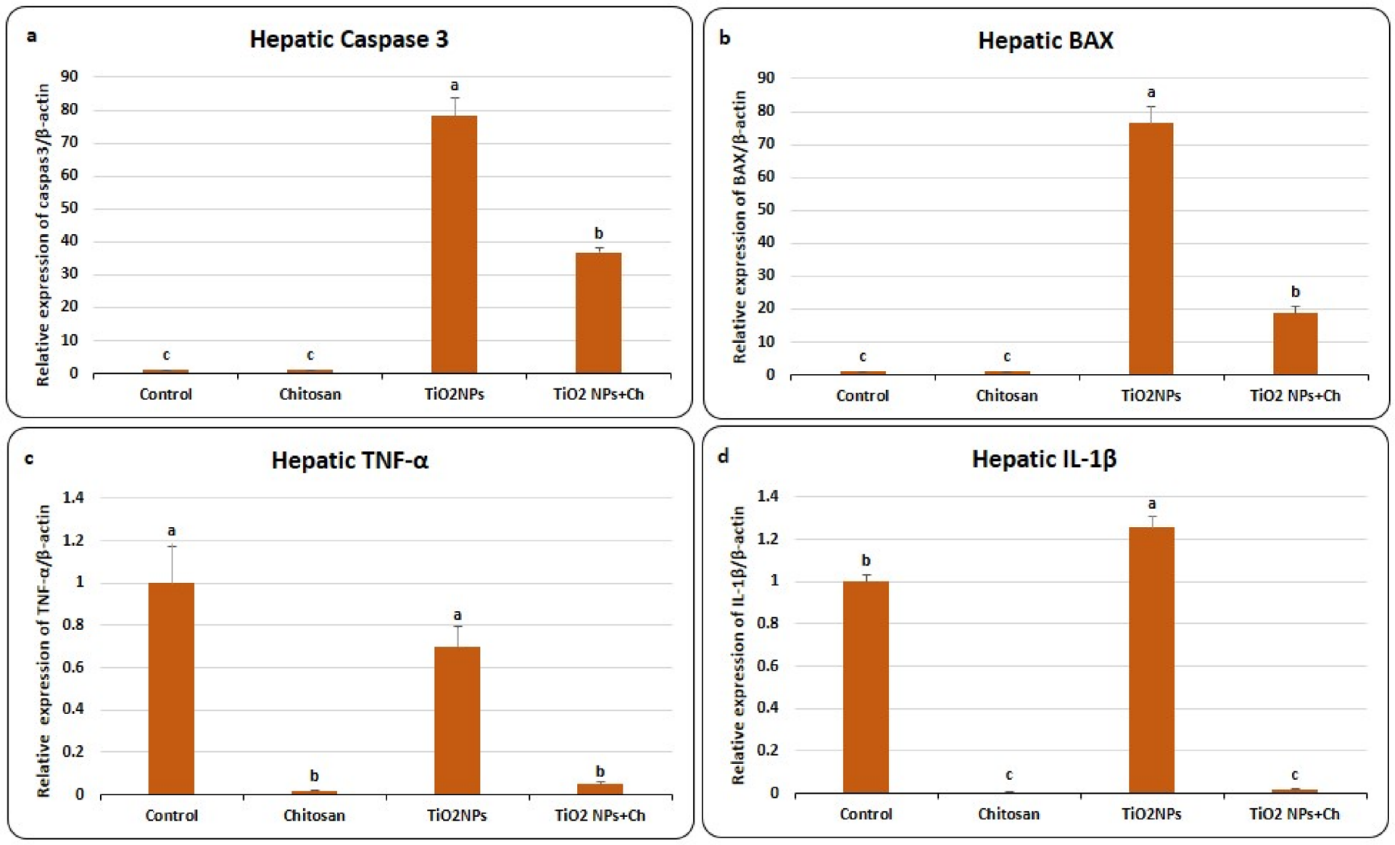
Effects of TiO_2_ NPs and chitosan on mRNA expression of hepatic genes encode apoptosis; Caspase 3 **(a)** and BAX **(b)** and hepatic genes encode inflammation; TNFα **(c)** and IL-1β **(d)**. Data were expressed as means ± SEM (*n* = 5). Bars with different superscripts means significant difference (*p* < 0.05).

As presented in Figure (2), nano titanium particles enormously upregulated mRNA expression of Caspase-3 (78.32 ± 5.55) compared to the control group (1 ± 0.069) (Fig. 2a). Chitosan treatment significantly mitigated the effect of TiO_2_ NPs (36.74 ± 1.27) in TiO_2_ NPs + Ch group compared to other groups. Likewise, relative expression of BAX was extremely increased in the TiO_2_ NPs group (76.47 ± 4.88) compared to the control group (1 ± 0.058) (Fig. 2b). In the TiO_2_ NPs + Ch group, chitosan significantly diminished the toxic effect of nano-titanium (18.81 ± 1.92) compared to other experimental groups. Single administration of chitosan exhibited no obvious effect on genes encoded apoptosis.

Regarding inflammatory genes, TiO_2_ NPs numerically down-regulated mRNA expression of hepatic TNF-α (0.7 ± 0.096) in comparison with the control group (1 ± 0.17) without significance (Fig. 2c). On the other side, chitosan significantly reduced the relative mRNA expression of TNF-α in the chitosan-treated group (0.017 ± 0.003) as well as in the TiO2 NPs + Ch group (0.048 ± 0.015) compared to the control one. Meanwhile, mRNA expression of IL-1β was significantly upregulated in the TiO_2_ NPs group (1.257 ± 0.053) compared to the control group (1 ± 0.029) (Fig. 2d). A significant reduction was observed in mRNA expression of hepatic IL-1β in chitosan (0.0057 ± 0.002) as well as TiO_2_ NPs + Ch (0.014 ± 0.004) groups compared to control and nano-titanium groups.

### Effects of TiO_2_ NPs and Chitosan on relative expression of renal genes encode apoptosis and inflammation (Fig. 3)

**Fig. 3:**
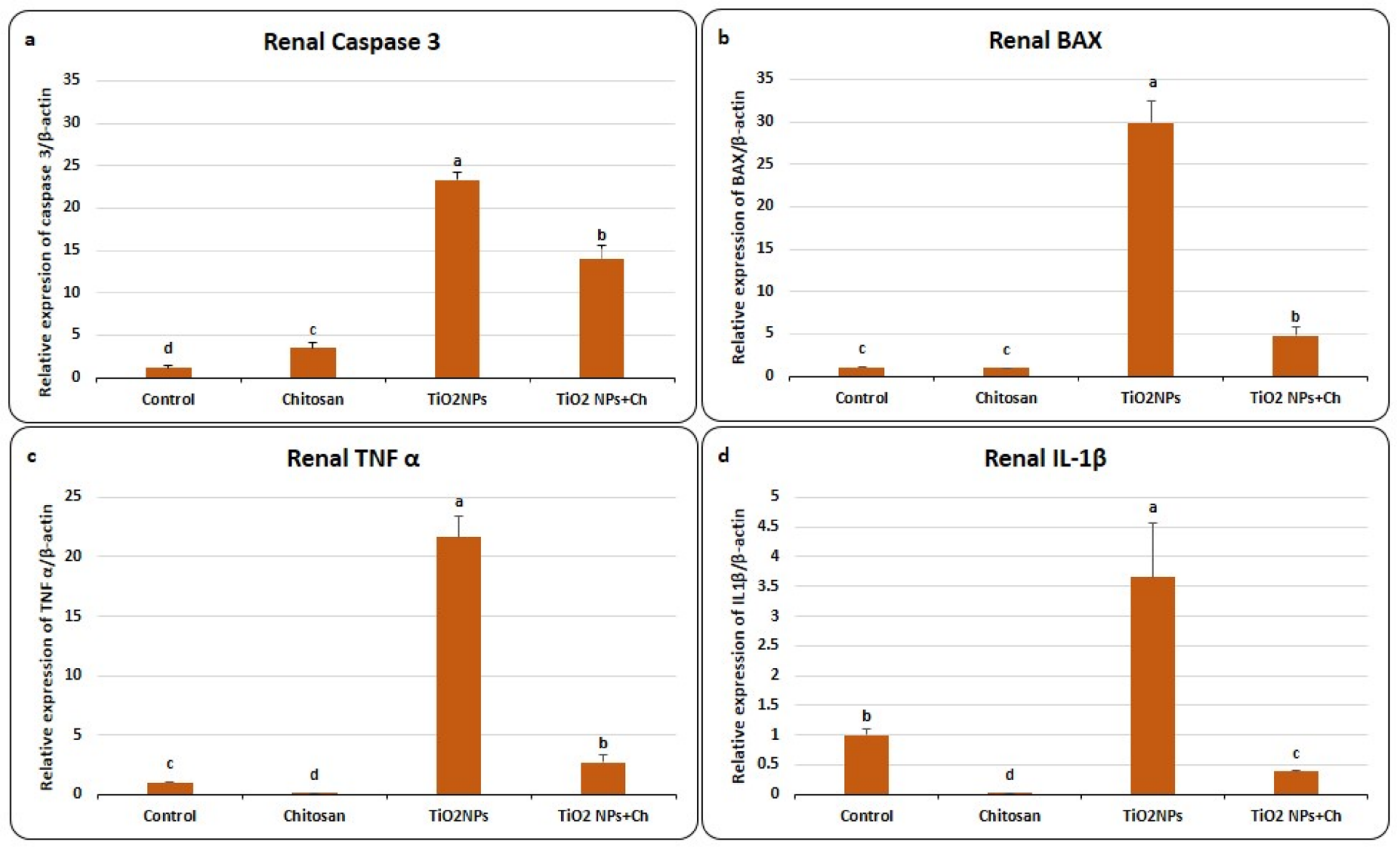
Effects of TiO_2_ NPs and chitosan on relative expression of renal genes encode apoptosis, Caspase 3 **(a)** and BAX **(b)** and renal genes encode inflammation; TNFα **(c)** and IL-1β **(d)**. Data were expressed as means ± SEM (*n* = 5). Bars with different superscripts means significant difference (*p* < 0.05).

As shown in Fig. 3, renal expression of Caspase-3 was significantly upregulated in TiO_2_ NPs group (23.29 ± 0.98) compared to control one (1.18 ± 0.21). Chitosan administration significantly reduced the toxic effect of nano-titanium in TiO_2_ NPs + Ch (13.95 ± 1.61) group compared to TiO_2_ NPs group (Fig. 3a). Similarly, relative expression of BAX was significantly increased in TiO_2_ NPs group (29.87 ± 2.56) compared to the control group (1.03 ± 0.05). While in TiO_2_ NPs + Ch group, mRNA expression of BAX was down-regulated (4.78 ± 1.02) compared to TiO_2_ NPs group (Fig. 3b). The mRNA expression of TNF-α was extremely induced in TiO_2_ NPs group (21.66 ± 1.75) compared to the control one (1 ± 0.086) (Fig. 3c). Chitosan treatment significantly mitigated the effect of chitosan in TiO_2_ NPs + Ch group (2.67 ± 0.63) compared to TiO_2_ NPs group. Similarly, mRNA expression of IL-1β was induced in the TiO_2_ NPs group (3.66 ± 0.9) in comparison with control (1 ± 0.1). In the TiO_2_ NPs + Ch group, expression of IL-1β was significantly down regulated (0.38 ± 0.02) compared to TiO_2_ NPs group (Fig. 3d). Chitosan significantly reduced relative expression of inflammatory genes in the kidney compared to the control group suggesting an anti-inflammatory action.

### Histological and morphometric evaluation

#### Histological and morphometric evaluation of liver (Figs. 4 and 5)

**Fig. 4:**
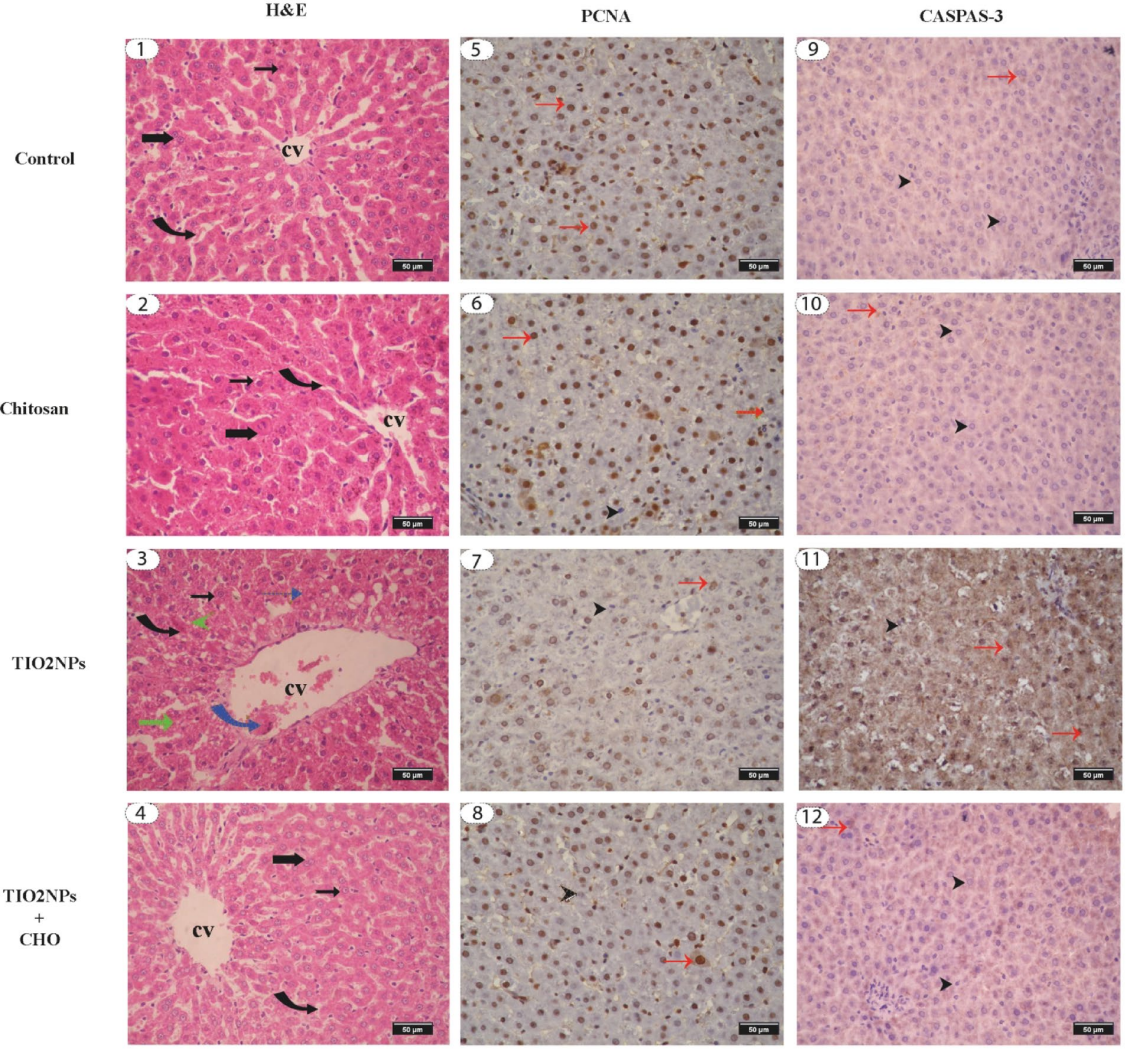
Photomicrograph of rat’s hepatic tissue stained with H&E stain in various groups (1–4) showing normal lobule with normal mononucleated hepatocyte (thin black arrow), binucleated hepatocyte (thick black arrow), normal central vein (CV), normal hepatic sinusiod (curved black arrow), abnormal hepatic cord (green arrow), congested central vein (curved blue arrow), pycnotic nucleus (blue arrow), congested blood sinusiod (green arrow head). The photos (5–8) represent enzyme immunohistochemical staining of hepatic tissue for PCNA showing normal strong positive reaction in normal hepatocyte (red arrow), negative reaction in some hepatocyte (black arrowhead). The photos (9–12) represent enzyme immunohistochemical staining of hepatic tissue for Caspase-3 showing normal negative reaction of hepatocyte (black arrow head). Strong positive reaction of Caspase-3 antibody in the hepatocyte (red thin arrow).

**Fig. 5:**
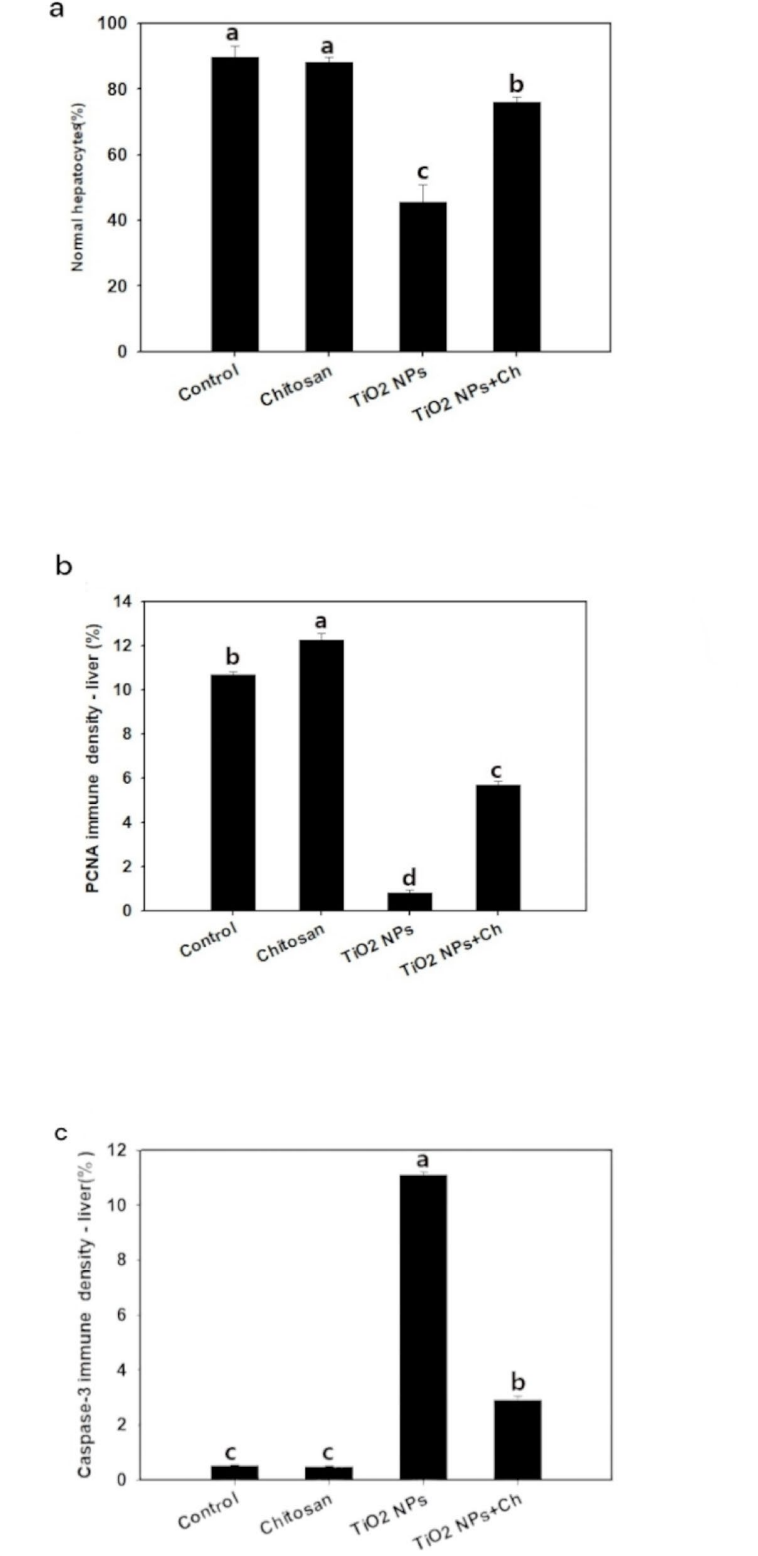
Morphometric analysis comparison among the different groups including: **(a)** Normal hepatocyte, **(b)** PCNA density in liver and **(c)** Caspase-3 density in liver.Data are presented as means ± SD. Bars with different superscripts mean significant difference (*p* < 0.05).

**Fig. 6:**
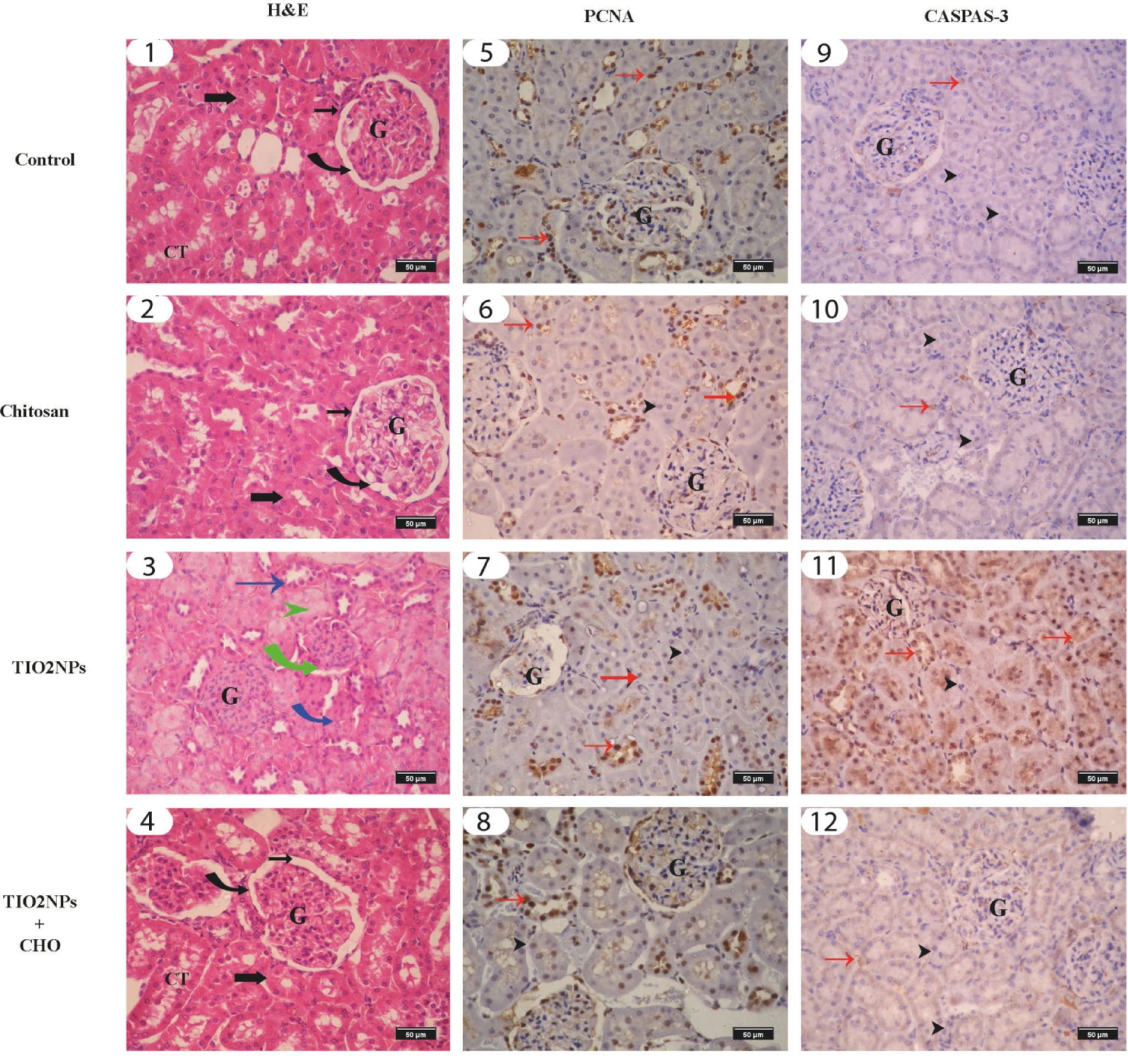
Photomicrograph of rat’s renal cortex stained with H&E stain in various groups (1–4) showing normal corpuscles (curved black arrow), parietal layer (thin arrow), glomerulus tuft (G), normal proximal convoluted (thick arrow) and collecting (CT) tubules, shrunken corpuscles (green curved arrow), coagulative necrosis (green arrow), inflammatory cells (blue curved arrow), tubular cast (green arrow head). The photos (5–8) represent enzyme immunohistochemical staining of renal cortex tissues for PCNA showing normal strong positive reaction of PCNA antibody in the normal corpuscles and renal tubular cells (red arrow). Negative reaction of degenerated renal tubule (black arrowhead). The photos (9–12) represent enzyme immunohistochemical staining of renal cortex tissues for Caspase-3 showing normal negative reaction of normal corpuscle and renal tubule (red arrow). Strong positive reaction of Caspase-3 antibody in the degenerated renal tubular cells (black thin arrow) and shrunken glomerulus.

Examination of liver tissues stained with H&E from the control and chitosan groups did not display observable differences and revealed normal histoarchitectures and cellular morphology (Fig. 4: 1,2) hepatocytes were normally arranged in cords separated by sinusoids. The hepatocytes were polygonal in shape and have abundant eosinophilic cytoplasm and central round or oval nuclei with normal lobular architecture and normal hepatocytes around the central vein, and the portal triad was unremarkable. On the contrary, sections from the TiO_2_ NPs group exhibited marked histological alteration in the liver (Fig. 4: 3) including, cellular alterations, degenerative hydropic changes and cellular infiltration in numerous hepatocytes, necrosis, congestion of sinusoidal blood vessels, disturbed hepatic architecture in most of the lobule and central vein congestion. In TiO_2_ NPs + Ch group, an apparent improvement in the histoarchitectures was observed (Fig. 4: 4) showing mild degree of degenerative changes in the hepatocytes. Mild congestion and mild vacuolar degeneration of hepatocytes. Additionally, the mean percent of the viable hepatocytes in TiO_2_ NPs group was significantly decreased (*p* < 0.05) compared to the control or chitosan group and the mean percent of viable hepatocytes with light vesicular nuclei and prominent nucleoli was significantly increased (*p* < 0.05) compared to TiO_2_ NPs group (Fig. 5: a).

#### Histological and morphometric evaluation of kidney (Figs. 6 and 7)

**Fig. 7:**
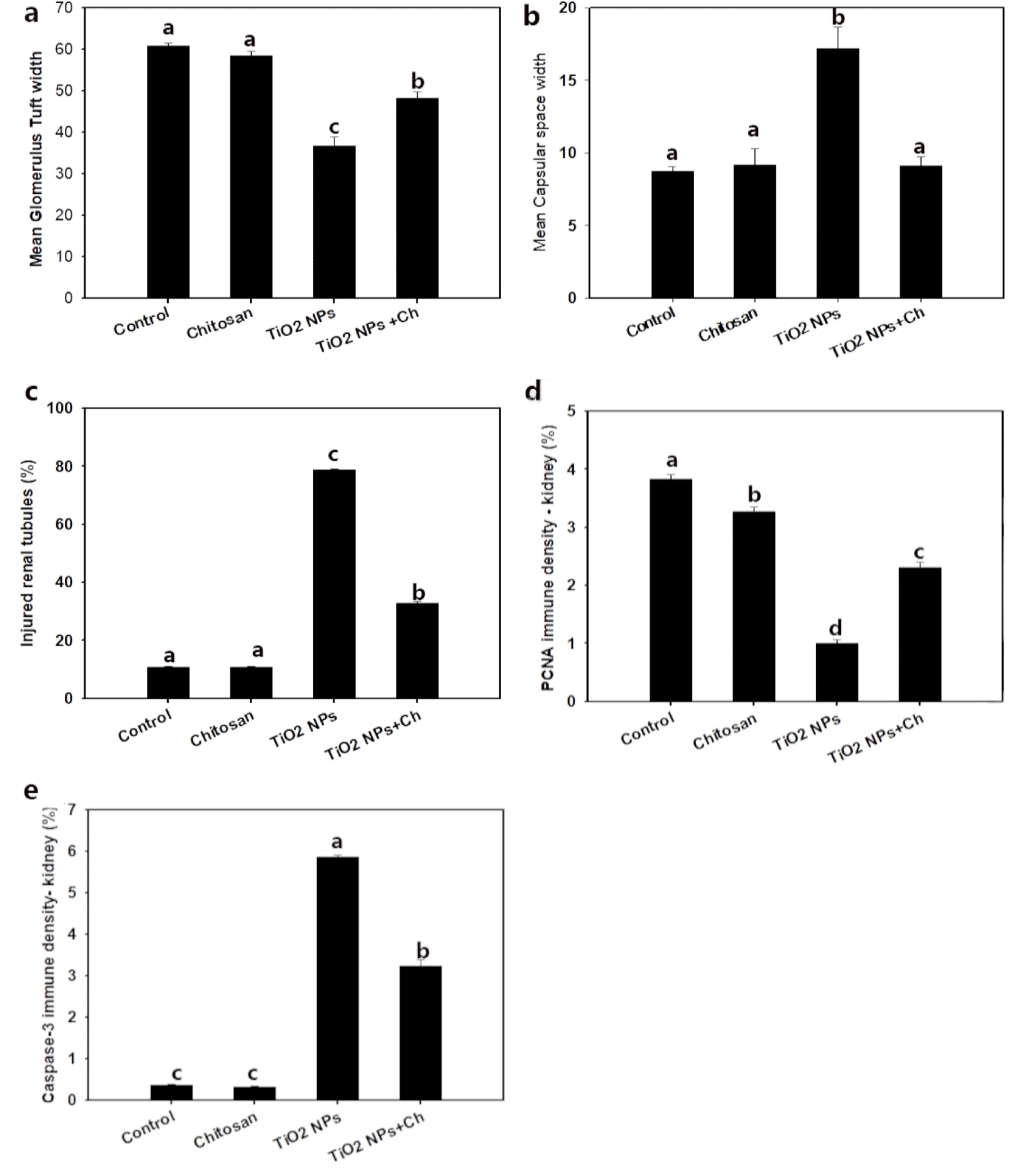
Morphometric analysis comparison among the different groups including: **(a)** glomerulus tuft width, **(b)** capsular space width, **(c)** injured renal tubule, **(d)** PCNA density in kidney and **(e)** Caspase-3 density in kidney. Data are presented as means ± SD. Bars with different superscripts mean significant difference (*p* < 0.05).

The histological photomicrographs of renal cortex sections stained with H&E from the control and chitosan groups (Fig. 6: 1, 2) revealed normal histological features of renal corpuscles, renal tubules had normal proximal and distal convoluted tubules, collecting ducts and interstitial tissue. The renal corpuscle was consisted of the outer parietal layer lined with simple squamous epithelium and inner visceral layer which supplies the glomerulus. Both the proximal and distal convoluted tubules were lined with cuboidal epithelium with a light vesicular nucleus but the cytoplasm of the distal convoluted tubule was lightly acidophilic and the lumen was wider than the proximal one. The collecting ducts had pale cytoplasm and were wider than proximal and distal convoluted tubules. In TiO_2_ NPs group, variable glomerular and tubule-interstitial changes were observed (Fig. 6: 3); Most of the epithelium of renal tubules showed hydropic degeneration in the epithelial cells of both proximal and distal tubules with pyknotic nuclei with exflotation of epithelium with extensive cellular debris and protenious accumulation (cast) in tubular lumen. Interstitial inflammatory cell infiltration was also observed. The preservation of renal corpuscles was obvious in TiO_2_ NPs + Ch group, an apparent improvement in the glomerulus tuft area and capsular spaces of this group (Fig. 6: 4).The glomerular tuft area was significantly increased in the TiO_2_ NPs + Ch group compared with the TiO_2_ NPs group (*p* < 0.05). The mean percent of the capsular space was significantly decreased (*p* < 0.05) in the TiO_2_ NPs + Ch group compared to the TiO_2_ NPs group (Fig. 7: a, b, c) and significantly reduced in the glomerular tuft area and injured renal tubule compared with control group (*p* < 0.05).

### Immune staining of PCNA and Caspase-3

#### Immune staining of PCNA and Caspase-3 in liver (Figs. 4 and 5)

Immunohistochemical determination of PCNA of hepatic tissue showed that expression of PCNA in the control group was quite evident in the nuclei of the hepatocyte (Fig. 4: 5). Chitosan administration increased the immunoreaction of PCNA and was evidently detected in the hepatocytes (Fig. 4: 6). The expression of PCNA was significantly decreased after TiO_2_ NPs administration (Fig. 4: 7). In the TiO_2_ NPs + Ch group, the immunostaining of PCNA was significantly increased (Fig. 4: 8) compared to the TiO_2_ NPs group and was near the PCNA immunoreaction in the control group.

Immunohistochemical determination of Caspase-3 of hepatic tissue revealed that the density of caspase-3 staining in both the control (Fig. 4: 9) and chitosan groups (Fig. 4: 10) showed negligible variation. In the TiO_2_ NPs group, the caspase-3 staining density was significantly increased (Fig. 4: 11). In the TiO_2_ NPs + Ch group, caspase-3 expression was significantly decreased (Fig. 4: 12) compared to the TiO_2_ NPs group. Morphometric analysis of Caspase-3 density among examined groups in liver was shown in Fig. 5: c.

#### Immune staining of PCNA and Caspase-3 in kidney (Figs. 6 and 7)

Expression of renal PCNA in the control group was quite evident in the nuclei of the normal corpuscles and renal tubular cells (Fig. 6: 5). Chitosan administration increased the expression of PCNA and was apparently detected (Fig. 6: 6). The expression of PCNA was significantly decreased after TiO_2_ NPs administration (Fig. 6: 7). In the TiO_2_ NPs + Ch group, the expression of PCNA was increased (Fig. 6:8) compared to the TiO_2_ NPs group and approached the PCNA expression in the control group. Morphometric analysis of PCNA density among examined groups in kidney was shown in Fig. 7: d.

Moreover, immunohistochemical determination of Caspase-3 of renal tissue displayed that the density of Caspase-3 staining in both the control (Fig. 6: 9) and chitosan groups (Fig. 6: 10) showed insignificant variation. In the TiO_2_ NPs group, the immune-reactivity of Caspase-3 was significantly increased compared to the control group (Fig. 6: 11). In the TiO_2_ NPs + Ch group, Caspase-3 expression was significantly decreased (Fig. 6: 12) compared to the TiO_2_ NPs group. Morphometric analysis of Caspase-3 density among the different groups in kidney was presented in (Fig. 7: e).

## Discussion

In the current work, TiO_2_ NPs did not affect relative weights of liver or kidney in experimental animals. At much higher dose, the significant differences were not observed in the coefficients of liver, spleen, and kidneys of the male mice received a single oral dose of TiO_2_ NPs (5 g/kg BW)^13^. Thus in the existing study, increasing relative organ weights was not expected during the experimental period.

In this study, oral titanium dioxide nanoparticles at a dose of 150 mg/kg BW (50–55 nm) induced hepatic and renal toxicities after 14 days of exposure in adult male rats. Serum levels of ALT, AST, ALP, urea, creatinine and uric acid were distinctly increased in the nano-titanium intoxicated group. Likewise, after 14 days slight increase in ALT/AST ratios was noticed after a single oral exposure to either 80–25 nm TiO_2_ NPs at a dose of 5 g/kg BW^13^. Both relative and protein expressions of AST, ALT and ALP were significantly up-regulated in the hepatic tissues of mice received TiO_2_ NPs at doses of 10 and 50 mg/kg BW for 60 days^14,15^. Moreover, daily intra-abdominal injection of TiO_2_ NPs (150 mg/kg BW) in female mice for 14 days exhibited augmentation in the activities of ALT, ALP and total protein, while uric acid and blood urea nitrogen levels were decreased with accumulation of nano titanium particles in hepatic and renal tissues^16,17^. Intragastric administration of TiO_2_ NPs (300 mg/kg BW) resulted in elevation of hepatic ALT, AST, ALP, and serum LDH representing liver injury^18^. In accordance, oral administration of TiO_2_ NPs (100 mg/kg BW) produced significant increase in total protein, ALP and AST, blood urea nitrogen and uric acid following a 14 days exposure period^19^. Subcutaneous TiO_2_ NPs (150 mg/kg BW) for four weeks on alternate days, displayed significant rise in ALT, AST. ALP, total bilirubin, urea and creatinine with a significant diminution in total protein^20^. Intraperitoneal TiO_2_ NPs increased ALT and AST levels, whereas blood urea nitrogen was not significantly affected suggesting that TiO_2_ NPs had a greater impact on liver than on kidneys^17^. It was reported that after intravenous injection of 5 mg/kg BW in rats, the levels of TiO_2_ NPs were higher in the liver than other organs^21^. Single intravenous injection of nano titanium at 300 and 645 mg/kg decreased direct as well as indirect bilirubin after 14 days, while uric acid was increased at 140 and 300 mg/kg BW^5^. Higher doses of intraperitoneal nano-titanium (4 and 16 g/kg BW) induced hepatic and renal damage via increases in AST and uric acid levels at 4 days and two months^22^. The liver is activated to remove the side effects induced by ingested TiO_2_ particles, and part of these particles should be expelled out by the kidneys. Nevertheless, the small size and difficult clearance of nano-titanium particles resulted in long-time retaining of them in vivo and fortified liver and kidney dysfunctions^13^. Nano-titanium particles induced morphological and physiological alterations in liver and kidney. In the liver, these alterations mainly affected the hepatocytes located around the centrilobular veins. The elevated liver enzymes as well as renal indices specified cellular leakage and damage of functional integrity of cell membranes owing to generation of reactive oxygen species which can peroxidize the unsaturated lipids of the cell membrane following depletion of antioxidant cell defenses that may produce oxidant/antioxidant imbalance^20^.

Administration of Carboxymethyl chitosan at a dose of 5 mg/kg BW improved hepatic and renal biomarkers in serum suggesting its optimistic influence on liver and kidney functions in rats exposed to nano-titanium particles. Likewise, following 7 days of treatment with chitosan (2.5, 5 and 10 mg/kg BW), there was a significant reduction in AST, ALT and ALP enzymes compared with that suffering carbon tetrachloride-induced hepatic fibrosis particularly at the highest dose^23^. The mechanism of its action was via inhibition of hepatic stellate cells activation, and inhibited the production of collagen 1 and 3.

Hepatic and renal oxidative stress as well as lipid peroxidation induced by nano titanium particles were due to the decline of SOD activity and GPx as well as to the increase of MDA levels^6^. Oxidative stress plays a serious role in the incidence of TiO_2_ NPs toxicity and is responsible for other cellular responses such as apoptosis, perturbation of cell cycle, and inflammatory response. A direct association was recognized among the oxidant stress induced by metal oxide nanoparticles, the interference of mitochondrial activity, and the induction of apoptosis via activation of Caspases-3 and − 9^24^.

In the current study, nano-titanium particles at a dose of 150 mg/kg BW reduced the activities of antioxidant enzymes with an augmentation in MDA in liver and kidney tissues, thus supported the hypothesis of oxidative stress involvement^25^. Similarly, intragastric administration of TiO_2_ NPs (300 mg/kg BW) induced lipid peroxidation and oxidative stress manifested by reduction in total antioxidant capacity, reduction in SOD, GPx and catalase activities^18^. Additionally, ROS production and GSH depletion were probably among the major causes leading to the apoptotic processes^26^. The large surface area of TiO_2_ NPs increased their capacity to produce ROS with further detrimental deviations and subsequent hepatic and renal toxicities^6,22^.

In the present study, TiO_2_ NPs up-regulated mRNA expression of TNF-α and IL-1β in both liver and kidney suggesting its ability to induce hepatic and renal inflammation. In consistency, a noticeable increase of hepatic TNF-α was observed in TiO_2_ NPs – intoxicated group at a dose of 300 mg/kg BW^18^. Serum levels of TNF-α were increased in mice receiving intragastric TiO_2_ NPs at doses of 10 and 50 mg/kg BW for 60 days^14^.

The appearance of inflammatory cells in liver tissue proposed that TiO_2_ NPs interacted with enzymes and other proteins in the hepatic interstitial tissue, interfered with the antioxidant defense mechanism resulted in production of reactive oxygen species that might induce an inflammatory response with dilated central blood vessel and blood sinusoids, as TiO_2_ NPs altered the permeability of hepatocytes membranes and the endothelial lining of blood vessels^20^.

Alternatively, chitosan exhibited noticeable anti-inflammatory effect in liver and kidney counteracting the toxic effect of TiO_2_ NPs primarily via its inhibitory effect on mRNA expression of TNF-α and IL-1β genes. Intraperitoneal chitosan at a dose of 400 mg/kg body weight reduced the levels of IL-1β and TNF-α and increased the expression of IL-10 in rats with cerebral hypoxic ischemia and in rats^27^. As well, chitosan nanoparticles at a dose of 20 mg/kg body weight exhibited renal anti-inflammatory effect in carbon tetrachloride-intoxicated rats following oral administration^28^.

Liver and kidney tissues showed severe diminution in PCNA immune density in TiO_2_ NPs group signifying the damaging impact of nano-titanium dioxide particles on hepatic and renal cellular proliferation^29^. Cells with positive immunostaining for PCNA means a state of tissue regeneration. While chitosan administration preserved PCNA levels in liver and kidney.

Reduction in PCNA levels were previously reported in kidney and lung damage induced by sepsis^30^, whereas Aflatoxin B1 as well as 2-nitropropane toxicity induced overexpression of PCNA inducing DNA damage in the liver and kidney tissues, this could be contributed to the carcinogenic effect of both toxicants^31–33^. Moreover, PCNA is an endogenous inhibitor of cell apoptosis, it interferes with apoptosis by binding procaspases, which prevents their activation and inhibits apoptosis^34^. Here, TiO_2_ NPs upregulated the relative expression of the pro-apoptotic molecules, Caspase-3 and BAX in hepatic as well as renal tissues. Up-regulation of BAX and Caspase-3 proposed induction of hepatic as well as renal apoptosis via the mitochondrial pathway. It was established that BAX induced apoptosis via stimulating the mitochondrial pathway, while Caspase-3 is activated in the apoptotic cell by extrinsic (death ligand) and intrinsic (mitochondrial) pathways^35^. In consistence, intraperitoneal injection of TiO_2_ NPs at doses up to 20 mg/kg body weight elevated the expression of Caspases-3 and 7 in liver and kidney of treated mice after 28 days of exposure^36^. Intraperitoneal TiO_2_ NPs at doses from 10 to 200 mg/kg body weight for 14 days induced hepatocytes apoptosis, damage in hepatic mitochondria, and generation of ROS in a dose-dependent manner. ROS might prompt inflammation and mutual feed-forward interaction between inflammation and oxidative stress with subsequent DNA damage and cell apoptosis^9^.

On the other side, administration of chitosan alleviated the adverse effect of TiO_2_ NPs via preservation of immune-staining of PCNA as well as reduction in the mRNA expression of Caspase-3 and BAX in TiO_2_ NPs + Ch group compared to individual exposure to nano-titanium particles, reflecting antiapoptotic properties of chitosan. In previous studies, chitosan nanoparticles inhibited apoptosis in cardiac cells through increasing expression of Bcl-2 and reducing expression of Caspase-3 in streptozotocin-exposed rats^37^. As well, chitosan nanoparticles down regulated 2-nitropropane- induced hepatic expressions of P53 and caspase-3 in Albino rats^31^.

The present study was the first that investigated the impact of chitosan against the destructive effects of TiO_2_ NPs in liver and kidney. Its protective effect versus lithium-induced oxidative stress and renal toxicity was previously established via lowering urea, creatinine and blood urea nitrogen levels at a dose of 200 mg/kg BW orally for 14 days^38^. Chitosan treatment reduced renal lipid peroxidation linked with stimulating the antioxidant system via increasing levels of SOD, catalase, GPx, and GSH in the renal tissue. Moreover, chitosan improved kidney architecture via counteracting the necrotic effect induced by lithium^38^. During induced-renal fibrosis in male mice, chitosan oligosaccharide (200 and 400 mg/kg BW/day by gastric lavage for 7 days) intervention not only increased SOD, GPx and GSH levels, but also declined MDA content in the obstructed kidney tissues in a dose-dependent manner, thus reducing oxidative damage^39^. Additionally, chitosan reduced serum levels of creatinine and blood urea nitrogen and inflammation with improving renal structural damage^39^. Chitosan nanoparticles at a dose of 5 mg/kg BW/day for 28 days reduced carbendazim toxicity via significant decrease in the serum levels of ALT, AST, creatinine, blood urea nitrogen and MDA, while catalase activity and total antioxidant capacity were increased^25^. Moreover, it improved the histological architecture of the liver as well as kidney tissues. Immunohistochemical staining showed diminishing in the positive percentage area of both immune markers, iNOS and Caspase-3, in hepatic and renal sections. Thus, chitosan nanoparticles exerted antioxidant, anti-apoptotic, and anti-inflammatory effects. Low molecular weight chitosan reduced serum creatinine and blood urea nitrogen levels in rats treated with 165 and 825 mg/kg/day for 13 days with improvement of renal tissue morphology^40^. Intraperitoneal chitosan nanoparticles (10 and 20 mg/kg orally for 2 weeks) afforded significant protection and amelioration against carbon tetra-chloride induced nephrotoxicity via reduction in serum creatinine and increase in the level of reduced glutathione with a significant decrease in MDA levels, TNF-α, IL-1β, and Caspase-3 concentrations at a dose of 20 mg/kg^28^. Silver nanoparticles-induced hepatic and renal damage was alleviated by chitosan nanoparticles at a dose of 140 mg/kg bw/day for two weeks, it exhibited significant decline in serum albumin, total proteins, calcium ions, GSH, catalase and SOD in renal and hepatic tissues^41^.

Administration of chitosan-nanoparticles (200 mg/kg BW/day orally for 12 weeks) ameliorated liver injuries induced by Diethylnitrosamine through increasing the activities of hepatic SOD, GSH, catalase concentrations and decreasing the values of biochemical parameters as ALT, AST, ALP, urea and MDA with increasing serum albumin^42^, suggesting the hepatic protective potential of chitosan nanoparticles as a powerful biomaterial and may be useful as antioxidants in hepatotoxicity^42^.

Promisingly, chitosan possessed a variety of beneficial biological effects, including anti-inflammatory, anti-oxidative, anti-diabetic, antihypertensive, and lipid-lowering activities^43^. Based on the histopathological investigation, it was noticed that TiO_2_ NPs caused various alterations in both liver and kidney architecture. In consistence, nano-titanium particles induced histological alterations in renal tissues in a dose (126, 252 and 378 mg/kg) and time dependent manner after 24 and 48 h post intraperitoneal injection in adult male rats^44^. The histological alteration showed renal damage represented by dilation and congestion in blood vessels with thickening in their walls and multiple large areas of hemorrhage. Additionally hepatic damage was observed as the hepatocytes appeared polyhedral with eosinophilic cytoplasm and having central rounded and vesicular nuclei with patent mononuclear cellular infiltration^20^. In consistency, histopathological analysis displayed that nano-titanium particles caused alteration in hepatic tissue including centrilobular necrosis, congestion and accumulation of inflammatory cells^10^.

It was suggested that high doses of TiO_2_ NPs might cause swelling of hepatocytes with obvious vacuoles in cells, and nuclear condensation, apoptosis and necrosis in hepatocytes. These findings supported that the mechanisms for hepatic toxicity of nano-TiO_2_ particles comprised mitochondrial damage and generation of reactive oxygen species resulting in expression disorders of protective genes^9^. Renal tissues of female mice in TiO_2_ NPs group (80 nm) showed serious swelling in the renal glomerulus and the renal tubule was filled with proteinic liquids. At the same study, hepatic tissues revealed prominent hydropic degeneration around the central vein and spotty necrosis of hepatocyte after two weeks exposure to TiO_2_ NPs^13^. Intragastric administration of TiO_2_ NPs triggered extensive histopathological alterations comprising dilatation of congested portal vein with RBCs, hydropic and vacuolar degeneration of hepatocytes beside edema and necrosis around dilated central vein, and inflammatory cell infiltration^18^. Distinct amelioration, with a significant decline in the pathologic lesion was verified in the group that received chitosan nanoparticles (20 mg/kg), in which renal tubules appeared normal in most of the examined sections, and mild vacuolation of the individual renal tubular epithelium was observed^28^.

## Materials and methods

This study was approved by Mansoura University Animal Care and Use Committee (MU-ACUC). The approval code was (R/50), Faculty of Veterinary Medicine, Mansoura University, Egypt. All methods were performed in accordance with the relevant guidelines and regulations following the ARRIVE guidelines.

### Chemicals

Titanium dioxide (TiO_2_) was purchased from Sigma-Aldrich Chemical Co., USA, and its nanoparticles, TiO_2_ NPs, were prepared at the Nanotechnology Unit, Faculty of Postgraduate Studies in Advanced Sciences, Beni-Suef University, according to Farghali et al. 2016^45^. The size range of nano-titanium dioxide particles is < 60 nm. Working solutions of dispersed TiO_2_ NPs were freshly prepared directly before administration via ultra-sonication for 15 min. Carboxymethyl chitosan (CMC, 10%) was purchased from Xin Luk Biotech, China.

### Experimental animals

Adult male Albino rats aged 3–4 months, were purchased from MERC lab and were kept for adaptation under standard laboratory conditions (temperature of 22–25 ⁰C, 50–60% relative humidity, and 12-hour dark/light cycle) for seven days. Food and water were offered ad libitum.

### Experimental design and treatments

Rats were randomly divided into four groups as the following:

- Control group: Rats received deionized water.
- Chitosan group: Rats administered chitosan at a dose of 5 mg/kg body weight according to Wang et al.^23^.
- TiO_2_ NPs group: Rats were given nano-titanium at a dose of 150 mg/kg body weight (1/80 LD_50_) according to Azim et al. 2015^46^.
- TiO_2_ NPs + Ch group: Rats received TiO_2_ NPs with chitosan at doses of 150 mg/kg BW and 5 mg/kg BW, respectively. All treatments were given orally for 14 consecutive days.

### Sampling

On the 15 th day, rats were weighed and euthanized by cervical dislocation. Blood was collected via cardiac puncture, centrifuged at 3500 rpm for 15 min for separation of sera and preserved in Eppendorf tubes at −20 ⁰C until further analysis. Liver and kidney tissue specimens were collected for biochemical, histopathological, immune-histochemical and molecular analyses. Relative weights of target organs were calculated according to Bearden and Fuquay, 1980^47^.

Relative organ weight = [Organ weight (g)/Bodyweight (g)]*100.

### Hepatic and renal tissue homogenates Preparation

Specimens from liver and kidney tissues were washed using cold sodium chloride solution (0.9%) and homogenized in a cold phosphate buffer saline (PBS, pH 7.5). Later, the homogenates were cold centrifuged for about 15 min at 3000 rpm and the supernatants were carefully collected in clean tubes for investigation of antioxidants and oxidative stress biomarkers^48^.

### Serum hepatic and renal biomarkers

Serum activities of alanine aminotransferase (ALT, REF; 20764957322), aspartate aminotransferase (AST, REF; 20764949322) (Roche Cobas Co., Indianapolis, USA), and alkaline phosphatase (ALP, catalog No.; A504-150) (Teco Diagnostics, USA), as well as, levels of total and direct bilirubin (BIOLABs, Maizy, France, REF; 80403), albumin (catalog No.; SB- 028–500) (Stanbio Laboratory, USA), total protein (catalog No.; SB-0250–500), creatinine (catalog No.; 10051) (Human Co., Germany), urea (catalog No.; URE118200) (BioMed Co., Cairo, Egypt) and uric acid (catalog No.; MD41001) (Spinreact Co., Santa Coloma, Spain) were estimated using spectrophotometer (Lambda EZ201; Perkin Elmer) according to the manufacturer’s instructions.

### Hepatic and renal antioxidant/oxidative stress biomarkers

The hepatic and renal levels of malondialdehyde (MDA, catalog No.; MD 25 29), catalase (catalog No.; CA 25 17), superoxide dismutase (SOD, catalog No.; SD 25 21), glutathione (GSH, catalog No.; GR 25 11), glutathione peroxidase (GPx, catalog No.; GP 2524), and glutathione-S-transferase (GST, catalog No.; GT 25 19), were estimated by spectrophotometer using commercial test kits bought from Biodiagnostics Co. (Cairo, Egypt).

### Quantitative real time PCR

#### RNA isolation and cDNA synthesis

Liver or kidney tissues were homogenized in Trizol™ reagent (100 mg/1 ml) (Invitrogen, UK) according to manufacturer instructions^49^. The concentration of RNA was determined using a nano spectrophotometer (Quawell, Q5000 UV-Vis spectrophotometer, San Jose, USA). An equivalent of 1 µg of RNA was transferred to cDNA with the High Capacity cDNA Reverse Transcription Kit^®^ (Applied Biosystems) using random hexamers in a 20 µl reaction volume that was further diluted 1:20 for further downstream analysis.

#### Quantification of genes encode apoptosis and inflammation using real-time PCR

Gene mRNA expression was measured by quantitative RT-PCR. Primers for genes that encode inflammation and apoptosis (http://www.ncbi.nlm.nih.gov/tools/primer-blast/*)* are listed in Table (4), including their sequences and accession numbers in Genbank (Table 4).

**Table 4:** Sequences of forward and reverse primers used for qRT-PCR quantitation.

Gene name	Primer sequence	Accession number
Caspase 3	F: GAATGTCAGCTCGCAATGGTAC R: AGTAGTCGCCTCTGAAGAAACTAG	NM_012922
BAX	F: AGACAGGGGCCTTTTTGTTAC R: GAGGACTCCAGCCACAAAGAT	NM_017059.2
TNF-α	F: ACTGAACTTCGGGGTGATCG R: CCACTTGGTGGTTTGTGAGTG	NM_001278601.1
IL-1β	F: TGCCACCTTTTGACAGTGATG R: AAGCTGGATGCTCTCATCAGG	NM_008361.4

The application of RT-PCR for amplification and relatively quantifying the definite genes in the present study was conducted on an Applied Biosystem Step One (Thermo Fisher Scientific, UK). Real-time PCR was accomplished using TOPreal qPCR 2x premix (enzynomics, South Korea) with the following cycling conditions: Initial denaturation at 95 ⁰C for 8 min, followed by 40 cycles of 95 ⁰C for 40 s, 56 ⁰C for 30 s, and 72 ⁰C for 40 s, then the reaction was terminated by a final elongation cycle at 72 ⁰C for 7 min. The expression analysis was applied using the 2ΔΔ ct method approved by Livak and Schmittgen^50^.

### Histological and immune-histochemical examinations

#### Preparation of specimens

Liver and kidney samples were fixed in 10% neutral buffered formalin solution for 24 h. Then, the tissues were gradually dehydrated with ascending ethanol concentrations, cleaned in xylene, and imbedded in liquid paraffin wax. Using a rotatory microtome, paraffinized blocks were sectioned (5 μm) and mounted on either coated glass slides for H & E staining or positive glass slides for immune-histochemical examination^51^. After staining, the slides were examined and photographed using an optical microscope^52^.

### Immune staining of proliferating cell nuclear antigen (PCNA) and Caspase-3

The technique was applied according to Petrosyan et al. 2002^53^. Briefly, liver and kidney sections were de-waxed, rehydrated, incubated with 3% H_2_O_2_ at room temperature for 30 min to inhibit endogenous peroxidase activity, and blocked with 5% normal goat serum for 15 min. Then, the sections were incubated overnight at 4 °C with primary antibodies against either PCNA (1:500, ab18197; Abcam) or Caspase-3 (1:100, 56046; Santa Cruz Biotechnology, CA, USA). Later, the sections were washed, incubated with secondary antibodies, stained with diaminobenzidine, and counterstained with hematoxylin stain. The mean density of PCNA and Caspase-3 expressions was evaluated and expressed as a percent using the image analyzer program (version 1.36, NIH, USA).

### Morphometric analyses

Using the light microscope (40X magnification), morphometric analyses were applied on randomly selected five stained slides for each group (5 fields/slide). The mean percentage of normal tissues was detected using the image analyzer program (version 1.36, NIH, USA). For hepatic tissues, the mean percentage of viable hepatocytes were counted. For renal tissues, five H&E stained sections were used to measure the glomerular tuft areas and the intra-capsular spaces, with a digital color camera attachment for studying the histopathological alterations. The percent of tubular damage was calculated and given the injury score from 0 to 5 according to Biswas et al. 2010^54^ as the following: score of 0 was given when there is no tubular injury, score − 1 indicated the tubular injury was mild and ≤ 10%, score − 2 indicated also mild tubular injury (10–25%), score − 3 was a moderate tubular injury (26–50%), score − 4 was an extensive injury (51–75%), and score − 5 was a severe injury (≥ 75%).

### Statistical analysis

The obtained results are reported, analyzed and presented as means ± standard errors. Statistical analysis for examined parameters was performed using the general linear model (GLM) produced by Statistical Analysis Systems Institute SAS, Statistical analysis system, SAS Procedure Guide, release 6.03 edition, SAS Institute Inc., Cary, Nc, U.S.A, 1998. Duncan’s New Multiple Range Test was used to test the significance of the differences between means^55^. Values of *p* < 0.05 were considered statistically significant.

## Conclusion

Collectively, after 14 days exposure period, TiO_2_ NPs provoked hepatic and renal toxicities in male rats that might negatively affect their vital functions. Whereas administration of chitosan alleviated its undesirable impact. The potential underlying mechanisms of chitosan to ameliorate TiO_2_ NPs-induced hepatic as well as nephrotoxicity were achieved by suppressing the oxidative stress, down regulating the mRNA expression of genes encode inflammation (TNF-α and IL-1β) and apoptosis (Caspase-3 and BAX) with reduction in PCNA and caspase-3 immune-density triggering antioxidant, anti-inflammatory, and anti-apoptotic effects. Therefore, chitosan could afford a prospective therapeutic system for the management of hepatic and nephrotoxicity.

## Data Availability

All data generated or analysed during this study are included in this published article.
